# Ethnoveterinary Practices of Medicinal Plants Among Tribes of Tribal District of North Waziristan, Khyber Pakhtunkhwa, Pakistan

**DOI:** 10.3389/fvets.2022.815294

**Published:** 2022-03-25

**Authors:** Sabith Rehman, Zafar Iqbal, Rahmatullah Qureshi, Inayat Ur Rahman, Shazia Sakhi, Imran Khan, Abeer Hashem, Al-Bandari Fahad Al-Arjani, Khalid F. Almutairi, Elsayed Fathi Abd_Allah, Niaz Ali, Muhammad Azhar Khan, Farhana Ijaz

**Affiliations:** ^1^Department of Botany, Hazara University, Mansehra, Pakistan; ^2^Department of Botany, Pir Mehr Ali Shah Arid Agriculture University, Rawalpindi, Pakistan; ^3^William L. Brown Center, Missouri Botanical Garden, St. Louis, MO, United States; ^4^Department of Botany, University of Swat, Swat, Pakistan; ^5^Department of Botany, Shaheed Benazir Bhutto University, Sheringal, Pakistan; ^6^Botany and Microbiology Department, College of Science, King Saud University, Riyadh, Saudi Arabia; ^7^Department of Plant Production, College of Food and Agriculture Science, King Saud University, Riyadh, Saudi Arabia

**Keywords:** ethnoveterinary practices, livestock, traditional medicine, Tribal Area, traditional knowledge, North Waziristan

## Abstract

Domestic animals play a vital role in the development of human civilization. Plants are utilized as remedies for a variety of domestic animals, in addition to humans. The tribes of North Waziristan are extremely familiar with the therapeutic potential of medicinal plants as ethnoveterinary medicines. The present study was carried out during 2018–2019 to record ethnoveterinary knowledge of the local plants that are being used by the tribal communities of North Waziristan, Khyber Pakhtunkhwa, Pakistan. In all, 56 medicinal plant species belonging to 42 families were identified, which were reported to treat 45 different animal diseases. These included 32 herbs, 12 shrubs, and 12 trees. Among the plant families, Asteraceae contributed the most species (5 spp.), followed by Amaranthaceae (4 spp.), Solanaceae (4 species), and Alliaceae, Araceae, and Lamiaceae (2 spp. each). The most common ethnoveterinary applications were documented for the treatment of blood in urine, bone injury, colic, indigestion, postpartum retention, skin diseases, constipation, increased milk production, mastitis, foot, and mouth diseases.

## Introduction

Plants have long been used as food (1), feed (2, 3), fiber (4), and shelter (5) by humans (6) and animals, as well as to control and alleviate diseases (7–9). Ethnoveterinary medicine (EVM) plays an essential role in animal production and livelihood development in many poor rural areas (10, 11) and is frequently the only option for farmers to treat their sick animals (12–14). The term “ethnoveterinary” is defined as “local people's beliefs and aboriginal knowledge and practice used for the treatment of animal diseases” (15–17). According to McCorkle and Schilihorn-van-Veen (7), it is a systematic study and application of indigenous knowledge for theory and practice in ethnoveterinary medicine (EVM). The information and ability of ethnoveterinary practices (EVPs) are recognized by means of experience and passed on verbally from one generation to the next (18, 19). Due to industrial and technological development, this local information remains in some parts of developed countries (20). Such knowledge and practices are passed down and retained from generation to generation (21), particularly by livestock owners. So, EVM is playing an important role in viable livestock farming in various parts of the world (22–24).

Pakistan is an agricultural country, with agriculture and livestock supporting up to 80% of the population (25, 26). Pakistan is the world's third largest milk producer, demonstrating the importance of cattle (27). Many Pakistani livestock producers are impoverished, and owing to financial restrictions (28), the majority of these farmers are unable to buy current allopathic medications, resulting in poor animal productivity and health. In such circumstances, ethnoveterinary medicine may be advocated as an alternative to contemporary pharmaceuticals (29), and it can aid in poverty reduction by enabling people to cure their animals using their own resources. Traditional indigenous medicine is still used in rural regions for human (30) and cattle diseases (31) and for the maintenance of excellent animal health in emerging nations, despite advances in the pharmaceutical industry and the creation of therapeutic agents (32–34). EVM knowledge, like all other traditional knowledge systems (35), is passed down orally from generation to generation and may become extinct (36) as a result of fast social, environmental, and technological changes (20, 37), as well as the loss of cultural legacy disguised as civilization (34). Traditional knowledge must be documented via systematic investigations (38–40) in order to be preserved before it is lost forever (21).

According to the literature, tremendous work has been done worldwide on the documentation of ethnoveterinary practices (8, 10, 18, 19, 41–43), but in Pakistan very little attention has been given to the documentation of EVM, resulting in limited reports on this important ethnoveterinary knowledge (12, 21, 44), revealing a significant gap in knowledge. For instance, Farooq et al. (45) reported 18 plant species representing 14 families to cure parasite disorders of livestock from the Cholistan desert of Pakistan, while Dilshad et al. (46) reported 66 plant species from Sargodha, Pakistan. Zia-ud-Din et al. (47) identified 35 plant species belonging to 25 families in a similar survey from the hilly area of Pakistan. Shoaib et al. (48) reported 41 medicinal plants belonging to 30 families to treat various livestock ailments from the Kaghan Valley, Western Himalayas–Pakistan. Siddique et al. (49) documented 80 medicinal plants belonging to 50 families to treat various livestock diseases from Haripur District, Khyber Pakhtunkhwa, Pakistan.

Various ethnoveterinary studies have been conducted in the allied areas of the study area (50–52), but no single ethnoveterinary documentation has been carried out in this unexplored, remote region of the tribal district of North Waziristan, Pakistan, highlighting the dire need to report this important knowledge. As a result, the current study is the first to investigate the entire ethnoveterinary practices of North Waziristan, Pakistan, where indigenous people have extensive traditional knowledge and rely heavily on medicinal plants to treat livestock ailments and supplement their income. This study might be beneficial to fill the gap that might have not been covered due to the negligence in documentation of ethnoveterinary practices. Therefore, it is extremely necessary to document and disseminate indigenous knowledge to help and share the different uses of plants as animal healthcare and to promote different conservation measures. Thus, the aim of this study was to evaluate and record the precious ethnoveterinary information of North Waziristan that is used by the area's indigenous inhabitants to treat domestic animals' diseases and disorders.

## Materials and Methods

### Study Area

**Figure 1:**
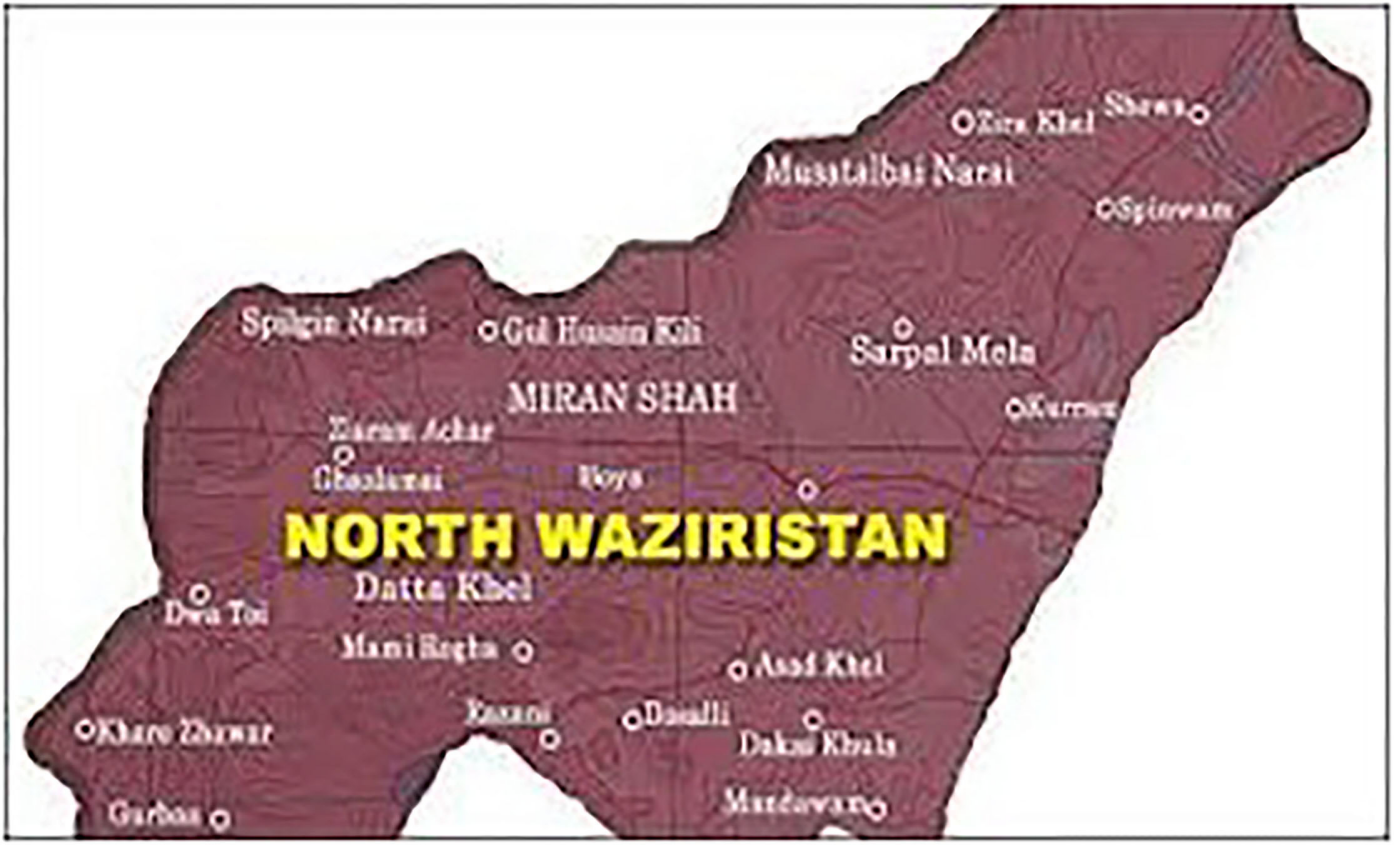
Map of the study area.

The tribal district of North Waziristan, Khyber Pakhtunkhwa, Pakistan, is a hilly region that lies between 32–35^°^ and 33–20^°^ north latitudes and 69–25^°^ and 70–40^°^ east longitudes with an altitude of 2143–7717 feet (Figure 1). North Waziristan is bounded on the south by the district of South Waziristan; on the north by Kurram Agency, Hangu District, and Afghanistan; on the east by the district of Bannu; and on the west also by Afghanistan. North Waziristan falls under the Irano-Turanian Region. The area is bounded by mountains, which are connected with Koh-e-Sulaiman in the south and Koh-e-Sufaid in the north. The tribal district is well-populated with various small dynamized villages. The main hills are Alexandra, Larema, Kalenjer Ser, Vezda, Ebulnki, and Sedgai Gher. The area is divided into three sub-divisions and nine tehsils. The Tochi Valley is 101 km long. The annual rainfall is 10“−13.” The summer period starts from May to September. The hottest month is June. The winter season starts from October to March. The coldest months are December, January, and February.

According to the census report of 2017, the total population of North Waziristan is 543,254. The total forest area is 475,000 acres. Wazir and Dawar are the major tribes in the research area. Pashto is the major language. The joint family system is practiced in the study area. The funeral and death ceremonies are mutually attended by the relatives and friends. The citizens of the area follow the jirga to determine their administrative and social problems. This is one of the strongest and most active common institutions in the area. The people in the area are mostly poor and earn their income from basic jobs. These include wood sellers, farmers, shopkeepers, horticulturists, local health healers, pastoralists, and government employees. In the study area, the domestic animals kept by the pastoralists are considered a better source of income.

### Field Survey and Data Collection

**Figure 2:**
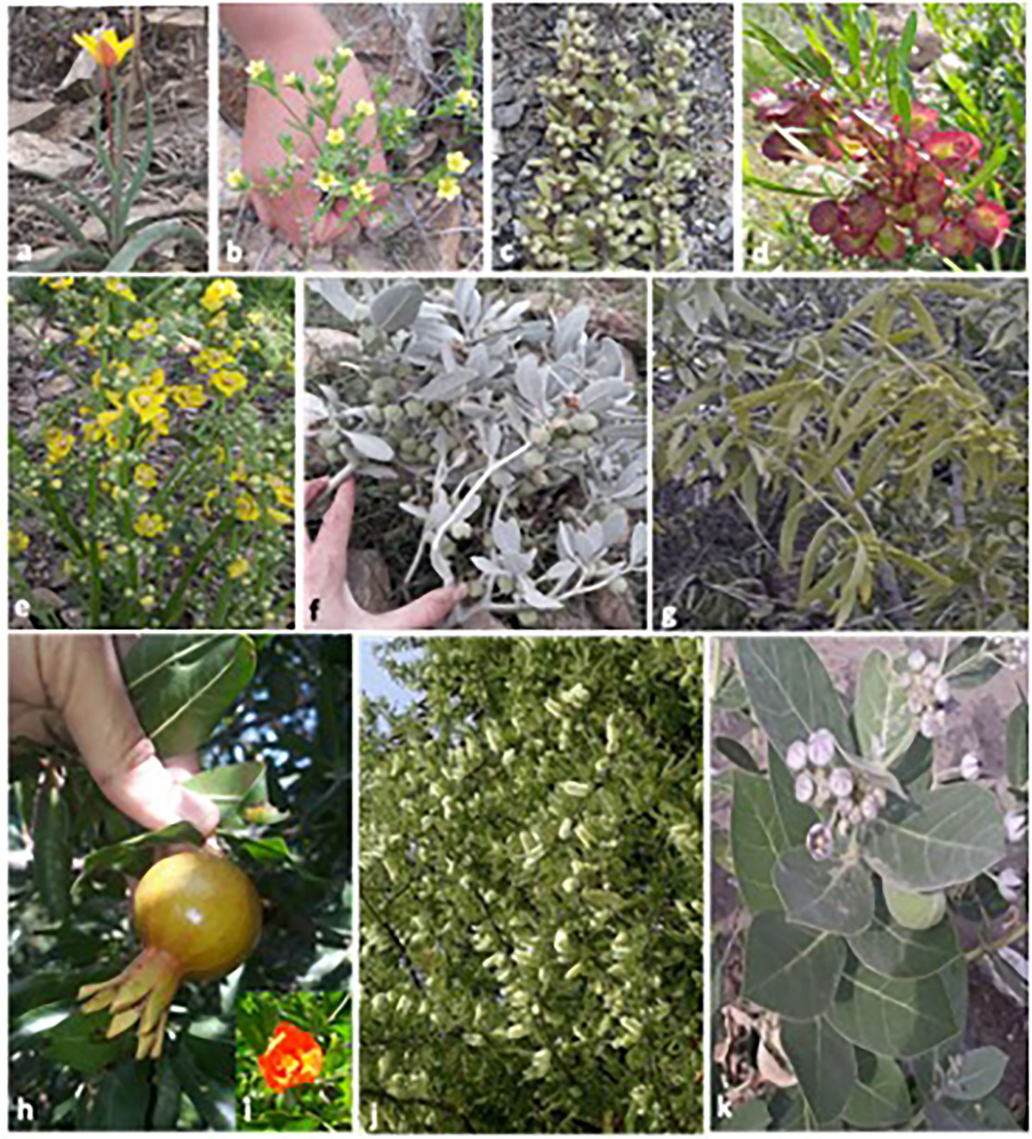
Photographs of some ethnoveterinary medicinal plant species of the study area.

The area of the study was visited during March and October 2018–2019 in order to collect ethnoveterinary data. The assessment was organized to collect data by using a semi-structured interview based on folk knowledge (35, 53) about plants that are used for the curing of various animal ailments (21, 54). The local people have valuable information about ethnoveterinary uses of the plants. All the collected plant species were photographed (Figure 2). During the study period, different types of herbal medicine were sold on the market, and the multi-use roles of some ethnoveterinary herbal plants were noted. Moreover, herbal remedy suppliers were interviewed. During the survey, every care was taken to note the vernacular names, dosage, parts used, mode of application, drug preparation method, and uses. Overall, 130 informants, 92 men and 38 women, were interviewed during the survey.

### Herbarium Work

Plant specimens were collected, pressed, dried, poisoned, and mounted on standard herbarium sheets (55, 56). The mounted specimens were then identified using published literature (57–59). For authentication purposes, Quaid-e-Azam University Islamabad, Pakistan, was also consulted. The identified specimens were deposited for future records in the Department of Botany at Hazara University, Mansehra, Pakistan.

### Statistical Data Analysis

The indigenous knowledge data were collected and analyzed statistically (36, 60, 61) using different quantitative indices: fidelity level (FL%) and informant consensus factor (ICF).

#### Fidelity Level (FL%)

The fidelity level (FL%) was the percentage of informants who reported the uses of certain plant species to cure a specific disease reported from the area of study. The fidelity level was calculated as follows (62, 63):


$$\text{FL}(\%)=\frac{\text{Np}}{\text{N}}\times \;100$$

where Np is the number of informants that mention a use of a plant species to cure a specific disease and *N* is the number of informants that use the plant species to cure any other disease.

#### Informant Consensus Factor

The informant consensus factor (ICF) was used to seek agreement among the informants on the documented cures for each ailment category (64).


$$ICF=\frac{\text{Nur-Nt}}{\text{Nur-1}}$$

where Nur is the number of use reports from informers in each disease category and Nt is the number of taxa used.

## Results

### Demographic Data

A total of 130 informants were interviewed. Most informants were men (83.85%) rather than women (16.15%). Many of them were over 60 years old (46.79%), 51–60 years old (39.23%), and 35–50 years old (23.88%). Due to the lack of educational facilities in that area, most of the informants were illiterate (45.38%; Table 1). But some were educated, showing that they had an awareness of education (8.46%). Many informants had completed primary (31.54%) and middle-level education (14.62%). All the informants spoke Pashto.

**Table 1:** Demographic details of the informants interviewed during ethnoveterinary survey in the study area.

**Gender**	**Number of informants *N* = 130**	**Percentage (%)**
Male	109	83.85
Female	21	16.15
**Age groups**		
35–50	17	13.08
51–60	51	39.23
>60 years	62	47.69
**Educational attainment**		
Illiterate	59	45.38
Primary	41	31.54
Middle	19	14.62
Secondary	11	8.46
**Social livelihood**		
Herbalists	38	29.23
Farmers	12	9.23
Shepherds	25	19.23
Gardeners	17	13.08
Local healers	28	21.54
Shopkeepers	6	4.62
Traders	4	3.08

### Ethnoveterinary Plant Species

During the present study, a total of 56 plant species belonging to 42 families were documented to be used in the treatment of 45 different ailments by the local herders, farmers, and shepherds in the tribes of North Waziristan, Pakistan. The results collected during the study are summarized, which provide the following knowledge for each plant species: botanical name, family name, vernacular name, habitat, part used, and disease treated (Table 2).

**Table 2:** List of ethnoveterinary plants (EVPs) used by tribes for healing of different ailments in North Waziristan, Pakistan.

**Sr** **#**	**Plant species**	**Family**	**Local name**	**Parts used**	**Habitat**	**Ethnoveterinary medicinal usage**
1	*Trianthema portulacastrum* L.	Aizoaceae	Deravenay Botay	Shoots	Herb	A shoot decoction is given orally to cows, sheep, and goats to expel abdominal worms.
2	*Allium sativum* L.	Alliaceae	Yeza	Bulb	Herb	The bulbs are crushed and mixed in wheat grain husk; the mixture is fed to cows and buffaloes 2 times a day to treat indigestion and abdominal pain.
3	*Allium cepa* L.	Alliaceae	Pyaz	Bulb, seeds	Herb	The seeds are given to birds and hens in case of twisting of neck and head (torticollis) disease. Crushed bulbs are mixed with brown sugar (Gur) and given to cows and buffaloes to treat fever.
4	*Aerva javanica* (Burm. f.) Juss.	Amaranthaceae	Gher valanai	Seed	Herb	The seeds (850 g) are mixed with 500 ml yogurt. The mixture is given 2 times a day for 12 days to cows, buffaloes, and goats for the treatment of blood in urine and diarrhea. Dry leaves (250 g) are ground into powder and mixed with 120 g hot butter oil. This mixture is given two times a day for 5 days to cows and goats to treat female sex organ infection.
5	*Amaranthus spinosus* L.	Amaranthaceae	Ghota surmi	Roots	Herb	A root decoction of about two cups mixed with brown sugar (Gur) is given to cows and buffaloes to increase the milk amount.
6	*Amaranthus viridis* L.	Amaranthaceae	Sormi	Leaves	Herb	Fresh plant leaves are crushed and mixed with pure ghee (butter oil). The paste is given twice a day for 3 days to goats, sheep, and cows to relieve constipation.
7	*Achyranthes aspera* L.	Amaranthaceae	Ghoskai	Leaves, root	Shrub	The juice made from the leaves of about one cup is given orally two times a day for 5 days to goats to remove urinary bladder stone. The fresh extract of the root is mixed with brown sugar (Gur), and 1 L mustard oil is given orally twice a day to buffaloes, cows, goats, and sheep to remove the placenta after birth.
8	*Trachyspermum ammi* L.	Apiaceae	Spirkay	Whole plant	Herb	A mixture of ajwain, black salt, and mint leaves at a ratio of 4:1:2 is given to cows, buffaloes, and donkeys to improve digestion. The whole plants are sliced and mixed with pickle and are given to goats, cows, and buffaloes to improve digestion. Seed powder of about 85–110 g is mixed with Gur. This mixture is given orally twice a day for 2 days to buffaloes and cows to expel the placenta.
9	*Arisaema flavum* (Forssk)	Araceae	Mangore botai	Rhizome	Herb	Roasted rhizomes are crushed and mixed with wheat flour and mustard oil and are given to cows and buffaloes for increasing milk yield.
10	*Arisaema jacquemontii* Blume	Araceae	Ghot mangore bote	Rhizome	Herb	One tablespoon of powdered rhizome is given orally with water to sheep and goats to expel intestinal worms.
11	*Calotropis procera* (Wild) R. Brown.	Asclepiadaceae	Spelmai	Latex, whole plant	Shrub	The fruits and leaves are crushed and are mixed with mustard oil and 90 g sulfur to form paste, and the paste is applied externally on cows and buffaloes to treat skin infection.
12	*Aloe vera* L.	Asphodelaceae	Zargya	Aerial parts	Herb	The fresh plant is crushed and put in drinking water to treat respiratory problems in goats, sheep, cows, and buffaloes.
13	*Artemisia martima* L.	Asteraceae	Terkha	Aerial parts	shrub	A 100-ml cup of decoction obtained from the aerial parts of the plants is given orally once a day for 3 days to cows and buffaloes for the killing and expelling of intestinal worms.
14	*Helianthus annuus* L.	Asteraceae	Ghurmasterge gul	Seeds	Shrub	The seeds are finely ground into powder, and the powders are given to hens with drinking water for 7 days to enhance egg yield.
15	*Sonchus oleraceus* L.	Asteraceae	Tareza	Leaves	Herb	Fresh plants are chopped and mixed with wheat grain husk and are given to goats, sheep, and cows to increase milk yield.
16	*Tagetes minuta* L.	Asteraceae	-	Shoots	Herb	About one cup of the concentrated infusion obtained from the shoots is given orally to buffaloes and cows to remove endoparasites like intestinal worms and liver flukes.
17	*Xanthium strumarium* L.	Asteraceae	Chechan botai	Leaves	Herb	Fresh juice extracted from the leaves is applied externally once a day for 3 days to kill larvae in wounds and to heal wounds.
18	*Berberis lycium* Royle	Berberidaceae	Nenakai boti	Bark	Shrub	Bark powder of about 250 g is mixed with butter oil and wheat flour. This mixture paste is given twice a day for 7 days to cows and buffaloes as body tonic, to treat internal fractures and strengthen the bone.
19	*Eruca sativa* Mill.	Brassicaceae	Khatel	Shoots	Herb	Young fresh shoots with leaves (4 kg) are boiled in 10 kg water to obtain an herbal tea. The herbal tea is mixed with 2 kg millet flour and is given orally to the horses 2 times a day for 5 days for the treatment of horse scabies.
20	*Bauhinia variegata* L.	Caesalpiniaceae	Kachnar	Leaves, flower	shrub	The paste made from crushed leaves and flowers is mixed with 5 g black salt and is given orally twice a day for 3 days to cows, buffaloes, goats, and sheep for the treatment of diarrhea.
21	*Cannabis sativa* L.	Cannabinaceae	Bhanga	Leaves	Herb	Powder made from leaves is mixed with black salt and is given orally once a day for 5 days to cows, buffaloes, and donkeys to increase appetite.
22	*Capparis decidua* (Forssk.) Edgew.	Capparidaceae	-	Tender branches	Tree	Tender branches are crushed to form fine powder. The powder is mixed with millet flour and given to cows and buffaloes to reduce their body pain and to improve the milk taste.
23	*Gymnosporia nemorosa* (Eckl. & Zeyh.) Szyszyl.	Celastraceae	Saghrzai	Leaves	Shrub	Infusions obtained from leaves of about 1 L are given orally twice a day for 3 days to cows and buffaloes for the treatment of diarrhea and dysentery.
24	*Convolvulus arvensis* L.	Convolvulaceae	Purvatia	Whole plant	Herb	The whole plant is crushed along with brown sugar (Gur), and the mixture is given to goats and sheep for 4–5 days to increase milk production and to treat constipation.
25	*Citrullus colocynthis* (L.) Schrad.	Cucurbitaceae	Marayghunye	Fruit	Herb	Powder (620 g) made from dried fruit is mixed with 130 g seed powder of *Withania coagulans*. This mixture is given 2 times a day for 7 days to horses and camels to treat rheumatoid arthritis.
26	*Cucumis melo* subsp. *agrestis* (Naud.) Grebensc.	Cucurbitaceae	Kukundai ghonde	Seeds	Herb	Whole seed powder of about 6–12 spoons is given to cattle and goats with drinking water to expel intestinal worms and liver flukes.
27	*Equisetum arvense* L.	Equisetaceae	Bandkai	Stem	Herb	Shoots (800 g) are crushed and mix with 1 L mustard oil. This mixture is given 2 times a day for 7 days to cows and buffaloes to treat urinary tract infection (blood in urine).
28	*Ricinus communis* L.	Euphorbiaceae	Arand	Leaves	Shrub	From the seeds, oil is extracted and is used as a laxative in animals. The extracts made from leaves are mixed with brown sugar. This mixture is given orally 2 times a day for 2 days to cows and buffaloes to speed up the expelling of the placenta.
29	*Indigofera heterantha* Wall.	Fabaceae	-	Young twigs, leaves	Shrub	The leaves and young twigs are given to cows and buffaloes to treat abdominal pain.
30	*Quercus incana* Roxb.	Fagaceae	Tora serai	Seeds	Tree	Powder (900 g) made from the seeds is mixed with 2 kg boiled millet and 1 kg brown sugar (Gur). This mixture is given orally once a day for 7 days to cows and buffaloes to increase milk production.
31	*Geranium wallichianum* D.Don.	Geraniaceae	Dhania ghonde	Roots	Herb	About 1–2 kg of roots is chopped, cooked, and mixed with 1 L of mustard oil. This mixture is given to cows, buffaloes, donkeys, and horses as body tonic.
32	*Mentha longifolia* L.	Lamiaceae	Zangali valanai	Roots	Herb	The root decoction (1 L) is mixed with half a liter of mustard oil and half a kilogram of brown sugar (Gur). This mixture is given to cows and buffaloes to increase the milk amount and to treat fever.
33	*Mentha spicata* L.	Lamiaceae	Serkare valanai	Leaves	Herb	Leaves (830 g) are crushed and mixed with 100 g black salt. This mixture is given orally to cows, buffaloes, goats, and sheep to improve digestion and to remove external parasites, e.g., anti-lice.
34	*Tulipa clusiana* DC.	Liliaceae	Shandai	Whole plant	Herb	The plants (2 kg) are chopped and mixed with 3 kg wheat grain husk. This mixture is given once a day for 12 days to cows, goats, and sheep to increase milk production.
35	*Linum strictum L*.	Linaceae	Showde tengavanai	Whole plant	Herb	Chopped plants (3 kg) are mixed with 3 kg wheat grain husk. This mixture is given once a day for 7 days to increase the concentration of milk.
36	*Melia azedarach* L.	Meliaceae	Bakana	Seed	Tree	Powder (500 g) made from the ripe seeds is mixed with 1 kg brown sugar (Gur). This mixture is given to cows and buffaloes as a lactagogue (promotes lactation).
37	*Acacia modesta* Wall.	Mimosaceae	Palosa	Bark	Tree	The bark decoction (500 ml) is mixed with 200 g butter oil. This mixture is given orally twice a day for 5 days to cows and buffaloes for easy parturition and for expelling the placenta.
38	*Morus alba* L.	Moraceae	Spin tooth	Fruit	Tree	Fresh fruit of about 3 kg is given once a day to cows, buffaloes, goats, and sheep to increase milk production and to treat constipation.
39	*Olea europaea* L.	Oleaceae	Zaiton	Fruit	Tree	The oil extracted from the fruits is given orally to animals for 4–7 days to treat indigestion and colic.
40	*Argyrolobium roseum* (Camb.) Jaub	Papilionaceae	Makhani booti	Shoots	Herb	The fresh plant (4 kg) is chopped and mixed with 2 kg wheat grain husk. This mixture is given once a day to cows, sheep, and goats to increase milk production.
41	*Cedrus deodara* (Roxb.) G. Don	Pinaceae	Almanza	Wood oil	Tree	The fresh heartwood is cut, and oil locally called “Ranzana” is extracted and applied externally on buffaloes to treat scabies and is also used as a mosquito and louse repellent.
42	*Plantago major* L.	Plantaginaceae	Ispaghul	Whole plant	Herb	The crushed plant material is applied externally on the affected hooves of cows and buffaloes for 6 days to treat mouth and foot disease locally called “Thabak.”
43	*Pennisetum glaucum* (L.) R.Br	Poaceae	Bajra	Seed		Mature grains (2 kg) are boiled in water and mixed with 1 kg brown sugar (Gur) and one cup of mustard oil. This mixture is feed to cows and buffaloes once a day to enhance milk production.
44	*Punica granatum* L.	Punicaceae	Velngai	Peel/husk	Tree	Fruits peels (700 g) are mixed with 500 ml curd. This mixture is given orally cows, buffaloes, goats, and sheep 2 times a day to treat dietary diarrhea. The outer fleshy part of the fruit is dried to make powder (600 g) and mixed in 500 ml curd. This mixture is given orally twice a day for 7 days to animal to treat kidney inflammation.
45	*Prunus armeniaca* L.	Rosaceae	Mondata	Leaves	Tree	Fresh or dry leaves are given to animal to treat constipation.
46	*Salvadora oleoides* Decne.	Salvadoraceae	Plawan	Fruit	Tree	Fresh fruit (2 kg) is given once a day for 12 days to cows, goats, and sheep in order to increase the milk concentration. Dried fruit (1 kg) is given once a day for 8 days to animals to treat rheumatism. Dried fruit (1 kg) is also given to cows and buffaloes after childbirth to make easy the removal of lochia (bleeding after childbirth).
47	*Dodonaea viscosa* (Linn.) Jacq.	Sapindaceae	Ghojara	Leaves	Shrub	The leaves of the plant are warmed and tied about 20–30 times to treat the fractured leg of animals.
48	Monotheca buxifolia (Falc.) A. DC.	Sapotaceae	Gurgora	Leaves	Tree	The plant leaves are orally fed to buffaloes and cows for 3 days to cure the unusual taste of milk; after 3–4 days, the original taste of the milk is retained.
49	*Verbascum thapsus* L.	Scrophulariaceae	Neshe botai	Leaves	Herb	Fresh chopped leaves (920 g) are mixed with 600 ml curd. This mixture is given orally 2 times a day for 5 days to cows, buffaloes, sheep, and goats to cure cough, diarrhea, and abdominal pain. Dried leaf powder is applied topically on wounds for quick healing.
50	*Datura alba* Nees.	Solanaceae	Berbaka	Leaves	Shrub	Warm leaves are applied externally to eliminate swellings from the animal body.
51	*Solanum melongena* L.	Solanaceae	Bengan	Fruit	herb	The fresh fruit is cut into minute parts and fed for 7 days to animals (cows, buffaloes) to boost female fertility.
52	*Solanum surattense* Brum. F.	Solanaceae	Korkendai	Fruit, leaves	Herb	Powder (850 g) made from ripened fruits is mixed with 500 ml honey. This mixture is given orally twice a day to cows, buffaloes, and horses to relieve abdominal pain. From the leaves and fresh fruit, poultice is made, which is applied externally to relieve pain in the animal body.
53	*Withania coagulans* (Stocks) Dun.	Solanaceae	Shafianga	Fruit, leaves	Shrub	From ripened fruits, 930 g powder is made and is given with drinking water once a day to sheep and goats to relieve abdominal pain. The powders of leaves are dissolved in water and are given to animals as a cooling agent.
54	*Tamarix aphylla* (L.) Karst.	Tamaricaceae	Ghez	Bark, leaves	Tree	The bark is finely ground to make powder, which is applied externally on animal skin to cure burning wounds. In animals the leaf smoke is used as an antiseptic after childbirth.
55	*Vitis negundo* L.	Verbenaceae	Marwandai	Leaves	shrub	Crushed leaves (800 g) are mixed with 90 g black salt and 350 g mint leaves. This mixture is given to cows, buffaloes, and donkeys to treat stomach problems and fever. A high dosage of dried leaves mixed with honey is used orally to expel worms in camels and cattle.
56	*Peganum harmala* L.	Zygophyllaceae	Sponda	Whole plant	Herb	The extraction made from the plant is externally applied on the animal body to kill lice (anti-lice). The poultice made from the leaves is externally applied to reduce pain from the crack bone in goats and sheep. The smoke from branches and leaves is used for 7 days to treat mastitis (breast inflammation) in animals.

### Habitat of Medicinal Plants

**Figure 3:**
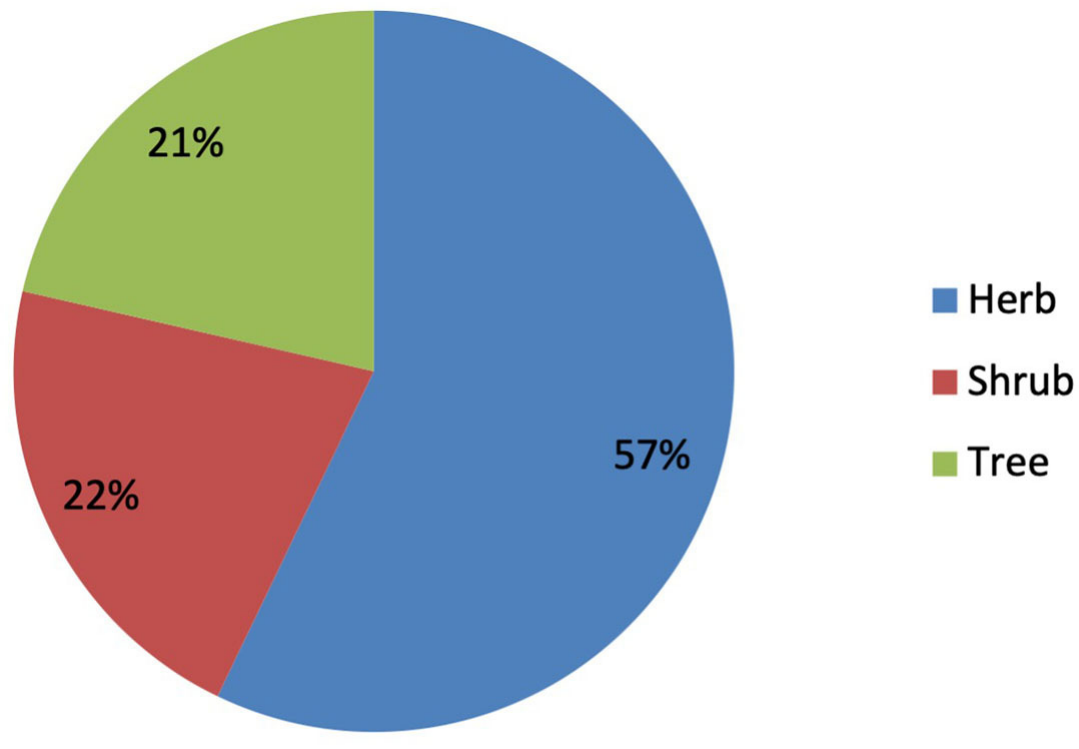
Growth form of the plant species reported from North Waziristan.

In the present study, it was revealed that the herbs were the most commonly used life form by the inhabitants, with 32 species (57.14%; Figure 3). It was followed by shrubs and trees (12 spp., 21.43% each). The dominant use of herbaceous plants in the study area in the preparation of remedies is due to profuse growth and easy availability in the wild as compared to other life forms.

### Medicinal Plant Families

The families that represented the highest number of plant species for the indigenous ethnoveterinary medicines were Asteraceae (5 spp., 8.93%), followed by Amaranthaceae and Solanaceae (4 spp., 7.14% each), Alliaceae, Araceae, Cucurbitaceae, and Lamiaceae (2 spp., 3.57% each), while the remaining families were represented by 1 species (1.79% each) in Table 2.

### Diseases Cured

Among all the 45 diseases in the study area, the indigenous healers and other local informants reported milk production (increased milk yield) as the most common problem treated through 12 plant species. It is followed by intestinal worms with 6 species each; abdominal pain with 5 species; constipation, diarrhea, and expulsion of placenta with 4 species each; and fever and indigestion with 3 species each. However, all the remaining 37 diseases were treated by <3 species each (Table 2).

### Plant Parts Used as Indigenous Medicine

**Figure 4:**
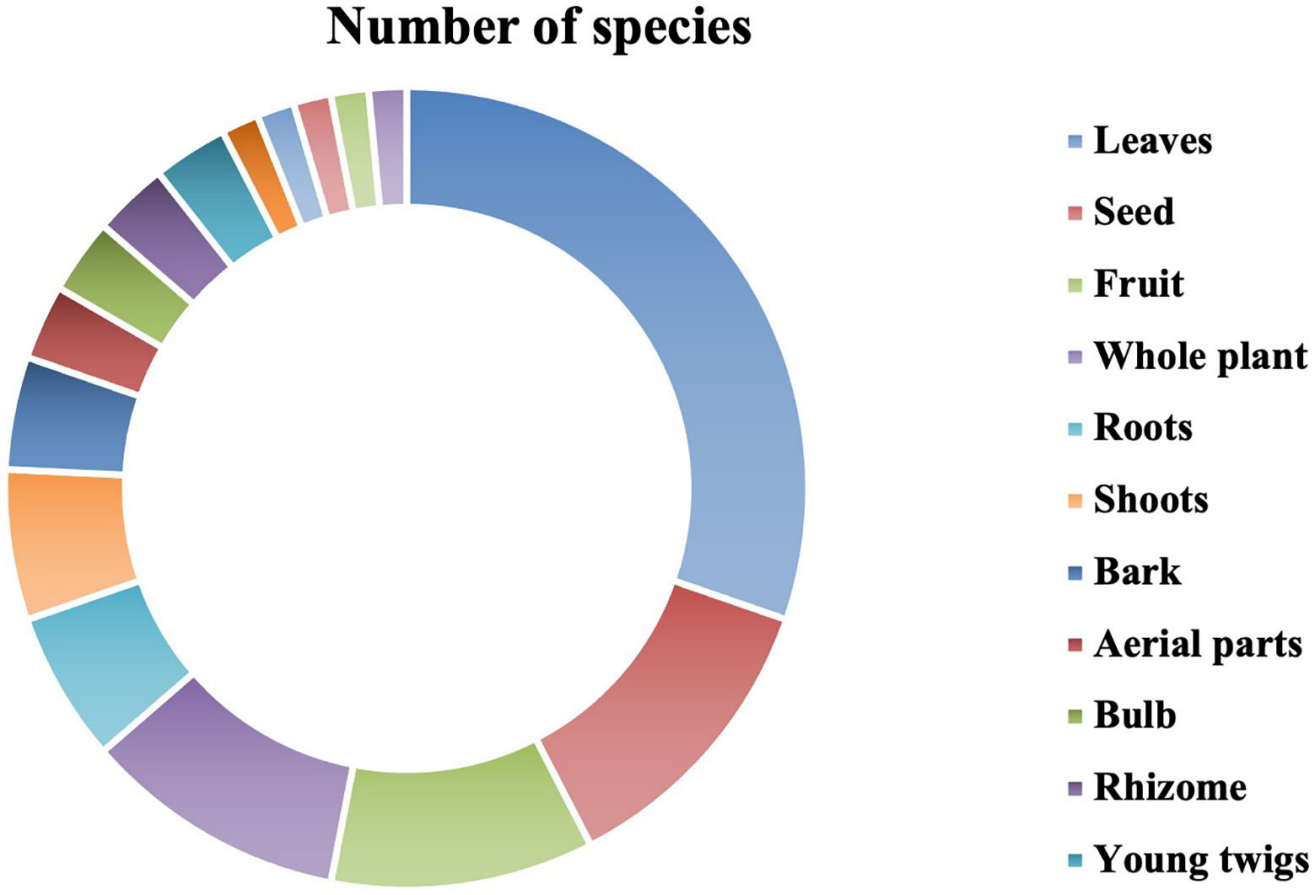
Plant species parts used in preparation of indigenous medicines.

Different parts of the plant were used to prepare ethnoveterinary medicine recipes to treat various ailments and are summarized in Figure 4 and Table 2. Out of 16 plant parts, leaves were heavily used to prepare medication (20 spp., 30.30%), followed by seeds (8 spp., 12.12%); fruits and whole plants (7 spp., 10.61%); roots and shoots (4 spp., 6.06%); bark (3 spp., 4.55%); aerial parts, bulbs, rhizomes, and young twigs (2 spp., 3.03% each); and flowers, latex, husk, stems, and wood oil (1 spp., 1.520% each). The collection of leaves and recipes for preparation from the leaves is much easier. So, leaves were the most used plant part in the preparation of remedies for the treatment of livestock ailments.

### Mode of Preparation in Indigenous Medicine

For treating 45 different diseases and ailments, about 11 different types of formulations were prepared from different plants (Table 3), in which most of the ethnoveterinary remedies were prepared as powders (16 spp., 25.81%), followed by raw (12 spp., 19.35%), crushed (9 spp., 14.52%), decoction and paste (4 spp., 6.45% each), and extract and infusion (3 spp., 4.84% each). Nonetheless, juice, oil, poultice, smoke, and wrapping (2 spp., 3.23% each), followed by the roasted mode of preparation for treating diseases, were recorded with the smallest number of species (1 spp., 1.61%). The most dominant method for the preparation of remedies was powder, which was used in the treatment of animals' diseases.

**Table 3:** Modes of preparation of the recorded species used in indigenous medicine.

**Mode of preparation**	**No. of species**	**Percentage (%) of species**
Crushed	9	14.52
Decoction	4	6.45
Extract	3	4.84
Infusion	3	4.84
Juice	2	3.23
Oil	2	3.23
Paste	4	6.45
Poultice	2	3.23
Powder	16	25.81
Raw	12	19.35
Roasted	1	1.61
Smoke	2	3.23
Wrapping	2	3.23

### Mode of Administration

In the current study, the most common mode of application/administration is oral application (48 spp., 81.36%), followed by topical application (9 spp., 15.25%) and inhalation (2 spp., 3.39%) (Figure 4). The majority of remedies were administered orally in the study area for the treatment of different ailments.

### Quantitative Analysis

#### Informant Consensus Factor

In the present study, we recorded ICF values ranging from 0.75 to 0.92 (Table 4). With 230 use reports, gastrointestinal disorder had the highest ICF value (0.92), followed by general health ailments (0.88); parasitic diseases (0.87); skeleto-muscular pain and inflammation (0.86); uterine disorders (0.83); analgesic, fever, and wound healing (0.82); dermatological disorders and antiseptic (0.80); urino-genital disorder (0.79); and respiratory diseases (0.75).

**Table 4:** Ailment categories and informant consensus factors or ICFs.

**Ailment categories**	**Biomedical term**	**Nt**	**Nur**	**ICF**
Analgesic, fever, and wound healing	Body pain, fever, wound healing, bone fracture, burning wounds	8	39	0.82
Dermatological disorders and antiseptic	Skin infection, scabies, antiseptic	4	16	0.80
Gastrointestinal disorder	Indigestion, increasing appetite, abdominal pain, constipation, colic, diarrhea, dysentery, stomach problems	19	230	0.92
General health ailments (GHAs)	Enhancing egg yield, increasing milk yield, increasing milk concentration, improving the milk taste, lactagogue	20	155	0.88
Parasitic diseases	Intestinal worms, larva in wounds, liver flukes, mosquito repellent, anti-lice	12	85	0.87
Respiratory diseases	Cough, respiratory problem	2	5	0.75
Skeleto-muscular pain and inflammation	Rheumatism, rheumatoid arthritis, mastitis, body swellings, torticollis, foot and mouth disease	6	37	0.86
Urino-genital disorder	Urinary bladder stone, blood in urine, kidney inflammation, urinary tract infection	4	15	0.79
Uterine disorders	Easy parturition, expel placenta, female sex organ infection, boost female fertility, lochia	8	43	0.83

#### Fidelity Level (FL%) of the Reported Ethnoveterinary Plants

In the present survey, the FL of medicinal plants varied between 40.91 and 100%. Four medicinal plants, viz., *Trachyspermum ammi, Salvadora oleoides, Peganum harmala*, and *Withania coagulans*, were the most important medicinal plants in the study area, which were particularly used to treat indigestion, lochia, mastitis, and abdominal pain, determined by 45 interviewers with 100% fidelity (Table 5), followed by *Berberis lycium*, which was the 2nd most important ethnoveterinary plant, having a fidelity level of 97.78% used in body tonic. *Aloe vera* was the third most important medicinal plant, having a fidelity level of 97.50% FL and used in respiratory problems, while *Capparis decidua* had the lowest fidelity level (40.91%) and is used in body pain. However, the rest of the medicinal plants were within the fidelity range of 41.38–95.56%.

**Table 5:** List of reported species with treated disorders and their fidelity level (FL%).

**Preferred species**	**Application**	**Np**	**N**	**FL (%)**
*Acacia modesta* Wall	Easy parturition	24	44	54.55
*Achyranthes aspera Linn*.	Urinary bladder stone	28	47	59.57
*Aerva javanica* (Burm. f.) Juss	Female sex organ infection	8	12	66.67
*Allium cepa Linn*.	Torticollis	11	20	55.00
*Allium sativum* L	Indigestion	9	15	60.00
*Aloe vera* L.	Respiratory problems	39	40	97.50
*Amaranthus spinosus L*.	Increasing milk amount	28	45	62.22
*Amaranthus viridis L*.	Constipation	25	46	54.35
*Argyrolobium roseum* (Camb.) Jaub	Increasing milk production	23	45	51.11
*Arisaema flavum (Forssk)*	Increasing milk yield	11	16	68.75
*Arisaema jacquemontii* Blume.	Expelling intestinal worms	17	29	58.62
*Artemisia martima* L	Expelling intestinal worms	38	45	84.44
*Bauhinia variegata* L.	Diarrhea	3	6	50.00
*Berberis lyceum Royle*.	Body tonic	44	45	97.78
*Calotropis procera (Wild) R. Brown*	Skin infection	13	14	92.86
*Cannabis sativa L*.	Appetizer	9	20	45.00
*Capparis decidua* (Forssk.) Edgew.	Body pain	9	22	40.91
Cedrus deodara (Roxb.) G. Don	Scabies	41	45	91.11
*Citrullus colocynthis (L.) Schrad*	Rheumatoid arthritis	27	38	71.05
*Convolvulus arvensis L*.	Constipation	43	45	95.56
*Cucumis melo subsp. agrestis (Naud.) Grebensc*	Expelling intestinal worms	12	23	52.17
*Datura alba* Nees.	Eliminating swellings	25	45	55.56
*Dodonaea viscosa (Linn.) Jacq*.	Fractured leg	28	40	70.00
*Equisetum arvense L*.	Urinary tract infection	40	47	85.11
*Eruca sativa* Mill.	Scabies	43	46	93.48
*Geranium wallichianum D.Don*	Body tonic	25	30	83.33
*Gymnosporia nemorosa* (Eckl. & Zeyh.) Szyszyl	Diarrhea and dysentery	40	46	86.96
*Helianthus annuus L*.	Enhancing egg yield	38	45	84.44
*Indigofera heterantha Wall*.	Abdominal pain	13	15	86.67
*Linum strictum L*.	Increasing the concentration of milk	24	37	64.86
Melia azedarach L.	Lactagogue	3	5	60.00
*Mentha longifolia* L	Fever	24	46	52.17
*Mentha spicata L*.	Indigestion	26	44	59.09
*Monotheca buxifolia (Falc.) A. DC*.	Unusual milk taste	37	45	82.22
*Morus alba* L.	Increasing milk production	28	55	50.91
*Olea europaea* L.	Colic	25	43	58.14
*Peganum harmala L*.	Mastitis	45	45	100.00
*Pennisetum glaucum* (L.) R.Br	Enhancing milk production	12	15	80.00
*Plantago major L*.	Foot and mouth disease	42	45	93.33
*Prunus armeniaca* L.	Constipation	34	45	75.56
*Punica granatum* Linn.	Kidney inflammation	31	43	72.09
*Quercus incana Roxb*.	Increasing milk production	14	23	60.87
Ricinus communis L.	Expelling the placenta	40	45	88.89
*Salvadora oleoides* Decne.	Lochia	45	45	100.00
*Solanum melongena* L.	Boosting female fertility	28	41	68.29
*Solanum surattense* Brum. F.	Abdominal pain	39	42	92.86
*Sonchus oleraceus* L.	Increasing milk yield	10	19	52.63
*Tagetes minuta* L.	Expelling liver flukes	12	23	52.17
*Tamarix aphylla* (L.) Karst.	Burning wounds	44	47	93.62
*Trachyspermum ammi* L.	Indigestion	45	45	100.00
*Trianthema portulacastrum L*.	Expelling abdominal worms	35	45	77.78
*Tulipa clusiana* DC.	Increasing milk production	12	29	41.38
*Verbascum thapsus* L.	Wound healing	23	43	53.49
*Vitis negundo* Linn.	Stomach disorder	21	39	53.85
*Withania coagulans* (Stocks) Dun.	Abdominal pain	45	45	100.00
*Xanthium strumarium* L.	Wound healing	6	11	54.55

## Discussion

One of the most important income sources for rural populations in the tribal district (North Waziristan) is livestock raising. According to the findings of the present study, farmers in the region rely on plants not only for food for their livestock but also for usage as medications to treat livestock diseases. In the present study, we have documented 56 plant species belonging to 42 families. Khan et al. (65) documented various animal diseases that were cured using indigenous herbal medicines. Similarly, Hassan et al. (34) reported 28 medicinal plants belonging to 23 families for treating various livestock diseases from Lower Dir District of Khyber Pakhtunkhwa, Pakistan. Badar et al. (66) recorded 46 medicinal plants belonging to 31 families for treating 26 various livestock ailments from the district of Jhang, Pakistan. Traditional remedies were little known to the younger generation, but the elders knew a lot more about how to cure livestock problems. These results are in accordance with Zerabruk and Yirga (67) and Khattak et al. (36). They found that the majority of respondents were elderly, with very few youngsters engaging in traditional livestock treatment.

The families that represented the highest number of plant species for the indigenous ethnoveterinary medicines were the Asteraceae (5 spp., 8.93%), followed by Amaranthaceae and Solanaceae (4 spp., 7.14% each). The higher distribution and richness of medicinal plant species from the aforementioned families might be linked to their dominance in the area (68). Furthermore, the widespread use of species from these groups might be linked to the existence of beneficial bioactive chemicals (69, 70) that protect livestock against diseases (71). Moreover, it was revealed that herbs were the most used growth form by the inhabitants, with 32 species (57.14%). They were followed by shrubs and trees (12 spp., 21.43% each). Herbaceous plants are also widely used in other parts of the world (72). The dominant use of herbs was also documented in other ethnoveterinary studies carried out by various researchers around the world (20, 21, 36, 37, 54, 73, 74). Indigenous healers used herbs most often as medication due to their availability in nature (75, 76), which are used by local people for the treatment of 45 animal diseases (blood in urine, bone injury, colic, indigestion, after-birth retention, constipation, milk production, foot and mouth disease, mastitis, and diarrhea, etc.). These diseases are usually observed in various animals, i.e., sheep, cows, goats, buffaloes, and horses.

The aboriginal people were collecting various plant parts (i.e., fruit, seeds, roots, bark, leaves, stems, aerial parts, and whole plants) used in the preparation of various remedies. Some important plants that are present but not in abundance are severely threatened due to maximum collection, trading, and grazing. The most frequently used plant parts are leaves (18, or 18.4% each), followed by seeds (6, or 6.1% each), fruits (6, or 6.1% each), whole plants (4, or 4.1% each), and roots and bark (2, or 2.0% each). Throughout the world, ethnic communities use leaves for the preparation of herbal ethnoveterinary medicine (77, 78). Similar findings were reported by Erarslan and Kultur (79); they reported that the local inhabitants of Turkey used leaves for most ethnoveterinary remedies. Poffenberger et al. (80) found that collecting leaves does not constitute a major threat to plant species' survival as compared to collecting underground parts such as stems, bark, or the entire plant. The usage of certain plant parts implies that these portions have the most therapeutic potential, although biochemical testing is required as well as pharmaceutical screening to double-check the location information (34). Traditional knowledge about indigenous ethnomedicine is mainly transmitted by oral tradition from generation to generation without any written record. Such practices are still common among rural and tribal communities in many parts of the world. Aged and uneducated people were more familiar with the preparation and use of remedies. The addition of scientifically validated ethnoveterinary applications in rural areas helps in poor quality improvement and raises livestock production (15, 81).

The common mode of application/administration is oral application (48 spp., 81.36%). The majority of remedies were administered orally in the study area for the treatment of different ailments. Similar results were also documented in previously reported literature (82). In the present work, some of the therapeutic properties of the plant species mentioned have already been validated based on pharmacology. For example, Kumar and Roy (83) prove practically that *C. procera* latex is used against inflammation. But the administration of ethnoveterinary recipes is a huge problem in the area because there is no standardized measurement unit for the plant remedies. Although the dosage was determined by using glass, cups, and plant parts like seeds and bulbs, the amount of dosage is usually dependent on the age, intensity of disease, and size of the animal. This type of dose measurement method is not appropriate. That is why veterinarians are dissatisfied with EVMs (84–86). The most dominant method for the preparation of remedies was powder, which was used in the treatment of animal diseases and showed similar results to the previously documented literature (87). However, the most common procedure of drug preparation, according to Deeba et al. (88) is grinding or crushing followed by soaking or boiling. In many cases, the procedure of medicine preparation differs from individual to individual person to the next. Traditional veterinary therapists preferred the same plant for treating the same disease in different ways (34).

In the present study, with 230 use reports, gastrointestinal disorder had the highest ICF value (0.92), followed by general health ailments (0.88) and parasitic diseases (0.87). The maximum number of informant citations for these infections indicates a high frequency of these ailments in the area. It has previously been reported that stomach disorders are more prevalent in lactating livestock, possibly as a result of poor feed and drinking water quality (89, 90). Medicinal plants utilized to treat disease categories with high ICF values are likely to have high potency, making them potential candidates for pharmacological and phytochemical research (91). The fidelity value of a medicinal plant species determines whether it is preferred for the treatment of a specific ailment (63). In the present survey, four medicinal plants, viz., *Trachyspermum ammi, Salvadora oleoides, Peganum harmala*, and *Withania coagulans*, were the most important medicinal plants that were particularly used to treat indigestion, lochia, mastitis, and abdominal pain, as determined by 45 interviewers with 100% fidelity. Moreover, *Berberis lycium* was found as the 2nd most important ethnoveterinary plant, having 97.78% FL and was reported as a body tonic, and *Aloe vera* was the 3rd most important medicinal plant, with 97.50% FL, used for respiratory problems. The maximum level of fidelity is always gained by widely utilized medicinal plants for specific ailments (90).

## Conclusion

This is the first ever study to document medicinal plants that are being used by the shepherds of North Waziristan, Pakistan, to treat livestock diseases. Due to modernization, the younger generations do not pay attention to the traditional knowledge of plants, and this knowledge was restricted only to shepherds, herders, farmers, and the elderly. Therefore, the present study is an important contribution to preserving the botanical wisdom of the local communities in treating animal diseases. The results showed that 56 medicinal plants are used in treating 45 diseases, of which 12 plant species are used in enhancing milk production. Among the highest uses of plants, five species, viz., *Trachyspermum ammi, Salvadora oleoides, Peganum harmala*, and *Withania coagulans*, were particularly used to treat indigestion, lochia, mastitis, and abdominal pain. Based on the results, it can be concluded that phytochemical and pharmacological screening of these plants is required to isolate the bioactive compounds, coupled with *in vitro* and *in vivo* studies for the reported veterinary diseases.

## Data Availability Statement

The original contributions presented in the study are included in the article/supplementary material, further inquiries can be directed to the first author.

## Author Contributions

SR collected the data. SR, RQ, and IR analyzed and interpreted the data and results. IR and SR wrote first draft of the manuscript. SS, IK, AH, A-BA-A, KA, EA, NA, MK, and FI helped in gathering literature and discussion section. AH, A-BA-A, EA, and FI helped in revising the manuscript. ZI and RQ supervised the work. All authors have read and approved the final manuscript.

## Funding

The authors would like to extend their sincere appreciation to Researchers Supporting Project Number RSP-2021/356, King Saud University, Riyadh, Saudi Arabia.

## Conflict of Interest

The authors declare that the research was conducted in the absence of any commercial or financial relationships that could be construed as a potential conflict of interest.

## Publisher's Note

All claims expressed in this article are solely those of the authors and do not necessarily represent those of their affiliated organizations, or those of the publisher, the editors and the reviewers. Any product that may be evaluated in this article, or claim that may be made by its manufacturer, is not guaranteed or endorsed by the publisher.
